# HOPES: An Integrative Digital Phenotyping Platform for Data Collection, Monitoring, and Machine Learning

**DOI:** 10.2196/23984

**Published:** 2021-03-15

**Authors:** Xuancong Wang, Nikola Vouk, Creighton Heaukulani, Thisum Buddhika, Wijaya Martanto, Jimmy Lee, Robert JT Morris

**Affiliations:** 1 Office for Healthcare Transformation Ministry of Health Singapore Singapore; 2 Institute of Mental Health Singapore Singapore; 3 Lee Kong Chian School of Medicine Nanyang Technological University Singapore Singapore; 4 Yong Loo Lin School of Medicine National University of Singapore Singapore Singapore

**Keywords:** digital phenotyping, eHealth, mHealth, mobile phone, phenotype, data collection, outpatient monitoring, machine learning

## Abstract

The collection of data from a personal digital device to characterize current health conditions and behaviors that determine how an individual’s health will evolve has been called digital phenotyping. In this paper, we describe the development of and early experiences with a comprehensive digital phenotyping platform: Health Outcomes through Positive Engagement and Self-Empowerment (HOPES). HOPES is based on the open-source *Beiwe* platform but adds a wider range of data collection, including the integration of wearable devices and further sensor collection from smartphones. Requirements were partly derived from a concurrent clinical trial for schizophrenia that required the development of significant capabilities in HOPES for security, privacy, ease of use, and scalability, based on a careful combination of public cloud and on-premises operation. We describe new data pipelines to clean, process, present, and analyze data. This includes a set of dashboards customized to the needs of research study operations and clinical care. A test use case for HOPES was described by analyzing the digital behavior of 22 participants during the SARS-CoV-2 pandemic.

## Introduction

We are at an age in health care where we have much data at our disposal, including the high penetration of digital electronic medical records and advanced techniques available for their analysis [1]. It is also well accepted that lifestyle characteristics, including activity, stress level, social interactions, and environment, are significant determinants of health outcomes [2,3]. Although estimates vary, it has been argued that *lifestyle choices* exceed the impact of *health care received* as a determinant of premature death [3].

It has been highlighted by Onnela [4] that the wide adoption of smartphones and the increasing use of wearable devices open up a new vista of characterizing both current health conditions and the ongoing behaviors that will determine how an individual’s health will evolve. As examples of these new data sources, we can readily measure physical activity, heart rate, heart rate variability, temperature, sleep, *sociability* (amount of human interaction), and smartphone usage (amount and duration of use, type of use, and the way a screen is tapped and scrolled). The approach of using personal digital devices to capture these data sources, and hence characterize an individual *in situ,* has been called *digital phenotyping* [5]. The use of digital phenotyping both complements and extends the use of traditional home monitoring (eg, blood pressure measurements) in telemedicine by offering continuous measurement during normal activities and everyday living. We have developed a general-purpose digital phenotyping platform called Health Outcomes through Positive Engagement and Self-Empowerment (HOPES), which integrates data from wearable devices and a broad set of smartphone sensors, provides an array of methods to inspect that data, and binds everything together into a platform with a comprehensive privacy and security model. The platform was developed in conjunction with a clinical study for schizophrenia. In what follows, we provide an overview of the HOPES platform and demonstrate its first use.

### Digital Phenotyping

The collection of data from a personal digital device can be used to encourage healthy behaviors; an example is the Singapore Health Promotion Board National Steps Challenge [6]. Data collection is sometimes combined with coaching or nudges for general wellness [7] or to monitor and improve an existing diagnosed condition [8,9]. We were originally inspired by the potential use of digital phenotyping to monitor and treat *mental health* conditions such as depression and schizophrenia. Several notable studies, including a study at Northwestern University, have shown the correlation of mobile phone sensors with depressive symptom severity [10]; a recent study at King’s College London showed the feasibility and acceptability of the extended use of wearable devices and smartphones in patients with schizophrenia [11] and the use of digital phenotyping for relapse prediction in schizophrenia [12]. The commercial world has also taken notice, and a number of start-ups have formed based on these technologies [13-15].

In addition, digital phenotyping is also being applied to address a diverse range of diseases, such as asthma, maternal health, cancer, and dermatology [9,16-18]. Recent experiments on the use of wearable devices such as the Oura ring [19] and the Fitbit wrist band [20] have been applied to measure participants’ parameters during the SARS-CoV-2 pandemic. We illustrate our observations related to the pandemic (using Fitbit) in the *Example Analysis* Section.

Digital phenotyping has the potential to supplement, and in some cases replace, standard clinical processes in data gathering and patient monitoring by virtue of the following attributes:

1. Productivity and cost: passive monitoring can be efficient for both the provider and patient, compared with traditional clinical visits or scheduled telemedicine encounters.
2. Latency: passive monitoring may enable relatively quick responses from health providers, for example, allowing for actions from a case manager within a day versus a week or longer for a visit.
3. Sensitivity: several variables such as resting heart rate or sleep parameters are not easy to measure in the clinic, and device monitoring can be more effective than subjective patient reporting or inconvenient manual processes. The emergence of low-cost consumer devices has been shown to be sufficiently accurate for several purposes [21].
4. More parameters: while in the past we have been limited to infrequent interview questions and scales, we now have the potential to monitor a wider variety of the subjects’ parameters, such as location, sleep, motion, heart rate variables, and instantaneous manual responses. These can be measured simultaneously, efficiently, and reliably.

A much-discussed concern is how well such techniques will be accepted and complied with by patients or consumer participants. This involves ease-of-use considerations by both the user and the provider. Another major concern revolves around data security and privacy preservation. These two aspects have been primary motivators in our design choices and investigations.

### Clinical Study on Digital Phenotyping in Schizophrenia

The HOPES platform was designed, developed, and refined concurrently to support clinical studies. The HOPE-S (Health Outcomes via Positive Engagement in Schizophrenia) study [22] was launched in November 2019. HOPE-S is an observational study of individuals with schizophrenia who were recently discharged from a psychiatric hospital. The aim of this study is to determine whether digital phenotyping data are associated with clinical and health utilization outcomes. Key events recorded over the 6-month observation period include readmission, outpatient nonattendance (ie, defaults), and unscheduled service use, such as emergency department attendance and mobile crisis team activations. The primary study outcomes are the ability to predict relapse and/or readmission within 6 months, with secondary outcomes being the associations between digital phenotyping data and health care use, psychiatric symptom severity, and functional status assessed during research visits. Ethics approval was granted by Singapore’s National Healthcare Group Domain Specific Review Board (reference no.: 2019/00720). To promote the use of digital wearables among our population and to provide incentives to patients to join our study, we offered each participant a Fitbit Charge 3 free of charge.

The first phase of the HOPE-S study is observational. During this phase, we examine the deployment, feasibility, and acceptability of a wide range of digital sensors while performing the analyses required to assess the outcomes described above. In this process, we have been collecting large amounts of data for our analyses. These data will subsequently be used to develop machine learning algorithms to *predict* changes in symptom severity and other important clinical outcomes, as opposed to merely analyzing associations. During the subsequent phase of the study, we will deploy interventions such as early warnings of relapses, which will allow pre-emptive steps to be taken to prevent participant relapse or rehospitalization.

### HOPES: A General-Purpose Platform for Digital Phenotyping

HOPES is based on, and extends, the existing *Beiwe* platform [23,24]. Our contributions include the following:

1. The integration of wearable devices, where we have experimented with both wrist and ring devices;
2. The use of a wide range of sensors on the smartphone.
3. An efficient onboarding method for participants.
4. A suite of user interfaces including data collection and quality management tools, clinical summarization dashboards, and general-purpose research dashboards for use in exploratory data analysis and building anomaly detection algorithms.
5. Assurances for data security and the preservation of user privacy.

The platform is designed to be reliably deployed at scale and makes use of both public clouds and controlled on-premise computing infrastructure. We recognize the broad spectrum of potential applications beyond mental health and the growing set of digital sensors and their capabilities that may be appropriate for different applications. Therefore, we designed HOPES to be flexible and extensible to accommodate new devices and sensor integration, and new data dashboards.

Although the data collected during the HOPE-S study are rich, they are also noisy and incomplete as is expected when dealing with real human behavior and varying data reliability among sensors. To address these challenges, we have developed a *data collection dashboard* and multiple data visualization and exploration tools, which have proven invaluable for monitoring and ensuring participant compliance on a daily basis in the research study. We have also developed a feature engineering pipeline to construct useful insights for the HOPE-S study and to compensate for various shortfalls in the raw data. These dashboards have been found to be easy to use by research coordinators involved in the HOPE-S study, who have been able to easily recognize problems and contact the participant if their data are not being received. We also illustrate the dashboards that our data scientists have used to look for patterns and an *anomaly detection dashboard* that raises alerts on irregularities in the data. All the data are then fed into downstream statistical analyses and our ongoing development of predictive machine learning algorithms. At this stage, our anomaly detection dashboard implements common statistical routines for anomaly detection in time-series data. The development of an effective relapse prediction algorithm is an ongoing subject of this study.

The remainder of this paper is organized as follows: In the section *Existing platforms* we review several existing open source digital phenotyping platforms, highlighting their respective strengths and weaknesses. In the section on *The HOPES Platform and Its First Use in the HOPE-S Study*, we describe the overall architecture of the HOPES platform. In the section on *Dashboards, User Iterfaces, and Data Analysis*, we describe the enhancements to *Beiwe* that the HOPES platform provides, guided by the requirements of the HOPE-S study and other planned future uses (including for purposes beyond mental health). In the section *Example Analysis* we show an early and simple example of the use of our collected data on 22 participants in which we compare user data before and after Singapore’s SARS-CoV-2 *lockdown* went into effect. In the section *Conclusions*, we provide some overall conclusions that can be drawn from our experiences with digital phenotyping.

### Existing Platforms

There are several existing open source digital phenotyping platforms, including *Beiwe* [23,24], *Purple Robot* [25-27], *AWARE* [28,29], and *RADAR-base* (Remote Assessment of Disease And Relapse) [30-32]*.* Each contains a core smartphone app that performs passive sensor data collection in the background and a server backend in charge of receiving the data. Note that digital phenotyping is not limited to smartphones; indeed, wearables also provide some significant differentiated capabilities, and there are other sources such as fixed detectors. Some platforms such as *Beiwe* and *RADAR-base* also support active data collection in the form of surveys and some capture data from wearable devices, such as wrist- or arm-wearable devices, by providing a common data interface.

From our assessment, Purple Robot has the most complete coverage of Android sensors and features among the platforms we reviewed. The user can select which sensors to turn on and set the data sampling frequency, however, the platform does not support the iPhone Operating System (iOS). AWARE supports both Android and iOS and has nearly full coverage of Android sensors and features. Similar to Purple Robot, AWARE also allows the user to configure sensors and features. RADAR-base has recently added iOS support and uses both passive (phone use and sensors) and active (survey and questionnaire) data collection. Although it covers fewer phone features and sensors than the Purple Robot and AWARE, it has a very attractive user interface and a very robust system for surveys and questionnaires. *Beiwe* is a smartphone-based digital phenotyping research platform that supports both Android and iOS and has a decent coverage of phone sensors and features. Moreover, the platform supports active feature collection from simple surveys. Apart from the data collection backend that receives data from participants’ phones, *Beiwe* also has a backend for data analytics.

We have based the framework for the HOPES platform on *Beiwe* for several reasons. First, *Beiwe* supports both Android and iOS, a requirement for any generic digital phenotyping platform to be widely adopted. Second, our platform analysis and comparison tests conducted in March 2019 showed that at that time, *Beiwe* was most ready to deploy. Our decision was also based on our review of a number of Git repositories and publications as well as previous practical applications of the platforms in clinical studies and trials. We chose the Fitbit wrist device to access data beyond the smartphone sensors after conducting a technical and usability comparison of several popular devices on the commercial market. Specifically, we compared Fitbit Charge 3, Huawei Honor A2, Xiaomi Mi Band 3, Actxa Spur+, and HeyPlus. We found that Fitbit was distinguished by ease-of-use, battery life, and reliability, and it has been validated to be reasonably accurate against gold standard devices for the measurement of sleep [21]. We also evaluated a number of external sleep measurement devices (such as mattress pads) but did not find them suitable for our purposes.

## The HOPES Platform and Its First Use in the HOPE-S Study

To support large-scale data aggregation of wearables, mobile phones, and other data sources, we defined a set of requirements and then built our platform to be secure and scalable. Building on top of the existing *Beiwe* platform, we created the HOPES platform by expanding the functional capabilities for easier participant onboarding, enhanced data collection monitoring, optimized data uploading, extended security features, expanded data processing and analytics pipeline, and a scalable deployment architecture. The goal was to obtain easy and secure onboarding, almost unlimited scaling, high operational security, and improved privacy assurance. Although we were immediately driven by meeting the strict requirements for the HOPE-S study, along the way we became aware of expanded requirements for a wider range of participant monitoring requirements. We took these requirements into account in our architecture and design, so we would be ready for further deployments. In this section, we describe the platform requirements, our resulting HOPES system architecture, the features collected for the HOPE-S study, the enhancements we made to the Android app, the platform backend, and the security protocols. We provide our motivation and a high-level description, leaving further details and information about miscellaneous improvements to Multimedia Appendix 1.

### Platform Requirements

The HOPES platform is designed to be a reliable, low-maintenance digital phenotyping collection and aggregation platform. It is designed to support research protocols as well as scale to larger production platforms, including self-service registration. The requirements and their corresponding capabilities are listed in Textbox 1.

To successfully implement such a broad set of requirements, we carefully studied and focused on the user experience for onboarding new participants and built a platform that leverages the best software engineering, design principles, and cloud architecture capabilities.

**Health Outcomes through Positive Engagement and Self-Empowerment (HOPES) platform requirements.**

**Requirements and implementation capabilities:**

Simple user onboarding

- Precreation of user identities and anonymization factors
- Preprinted Quick Response code onboarding sheets
- Ability to migrate participants to new phones (if their current phones are not usable for a study) while maintaining study data integrity and privacy
- Simple onboarding literature and packaging in *gift pack* format
- Wide platform support, Android (with and without Google services), iPhone Operating System
- Ability to be totally passive with zero user interaction after setup
- Preparation for self-service onboarding in the future

User data collection and privacy

- All data deidentified (no personally identifiable information)
- Per-participant encryption keys
- Per-participant random credentials
- Mapping between participant ID and deidentified study ID securely retained but only made available to authorized clinicians
- Secure data backup and archiving

Data security end-to-end

- Data encrypted while in cloud storage environment
- Data decrypted, but still deidentified and obfuscated where appropriate, in data analytics pipeline on-premise

Wearable support that is scalable and secure

- Precreation of wearable device accounts
- Wearable cloud accounts deidentified using study ID
- Wearable data automatically encrypted with user’s password
- Server-less functions to periodically collect and archive user data

Infrastructure, scale, and operational security

- Two-factor authentication for all participants, including certificate and credential authentication
- Rotating credentials
- Data collection dashboard
- HOPES work/ticket queue for monitoring alerts/logs/events
- Distributed Denial-of-Service and web application firewall protection
- Elastic scale at all levels
- Isolation of functions across private virtual private local area network
- Separation of administrative and data upload interfaces
- Private virtual private network for administration
- Separation of data upload application programming interface and data management
- Restricted access controls
- Automated repeatable deployment

Data analytics

- Data downloaded on-premise into secure workspace for analytics or clinical use
- Multistage analytics processing pipeline
- Anomaly detection dashboard
- Data exploration dashboard

Security standards

- Secure development process
- Automated patching
- Additional requirements from Singapore security and Information Technology standards

Expanded data collection support for social media metadata

- Support for deidentified metadata for WhatsApp text and audio/video messages on Android
- Phone text messages

Study clinical support for easy clinical management

- Daily deidentified data collection dashboard emailed to study researchers and clinicians to monitor study compliance
- Encrypted deidentified clinician dashboard accessible to clinicians

### Overall System Architecture

**Figure 1:**
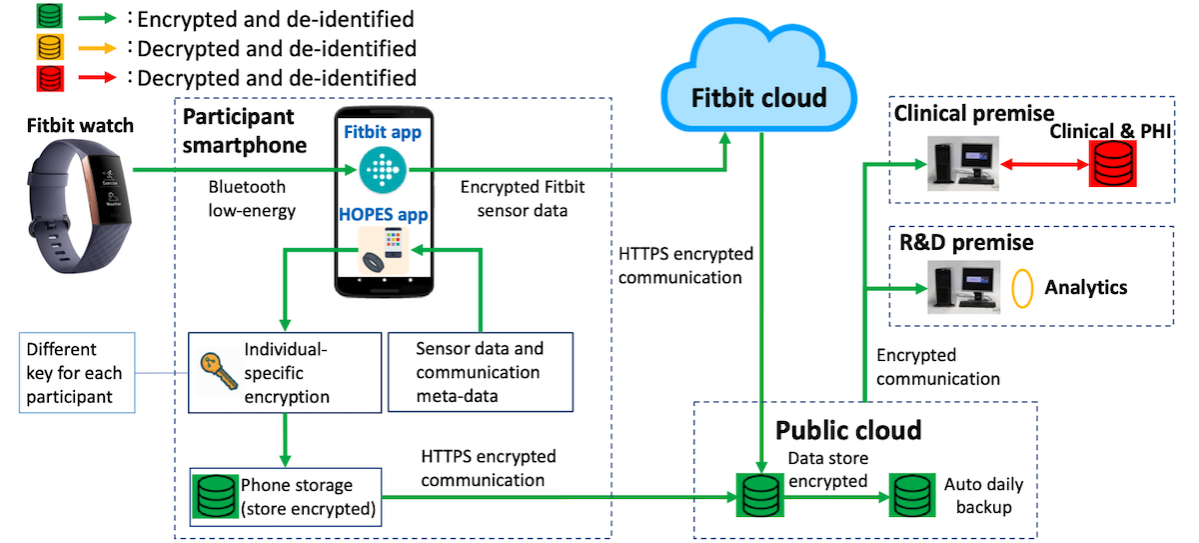
Overall system architecture and data flow diagram for the Health Outcomes via Positive Engagement in Schizophrenia (HOPE-S) study.

The high-level solution architecture of HOPES, as used in the HOPE-S study, is shown in Figure 1. On each participant’s smartphone, we installed two apps: the Fitbit app and the HOPES app. Every participant was required to wear a Fitbit watch for a certain portion of the day and night (enough to collect the required data but also allowing removal for charging, showering, etc). Fitbit raw data are collected by the Fitbit app and sent to a Fitbit server (the *Fitbit Cloud*) for processing and computation of high-level features (eg, the estimation of sleep stages). Phone data are collected on the smartphone by the HOPES app and sent to *a data upload server* hosted in a public cloud using Amazon Web Services (AWS). The *data processing backend server,* located at either the Research and Development (R&D) or clinical premises, periodically pulls data from both the Fitbit cloud and the AWS *data upload server* for subsequent processing, as described in the following sections. The data are always deidentified when in a publicly accessible cloud environment, and all transmissions and storage are encrypted. Certain variables, such as location, are also obfuscated at the time of collection for privacy preservation. More details on the solution architecture are provided in the Multimedia Appendix 1.

**Figure 2:**
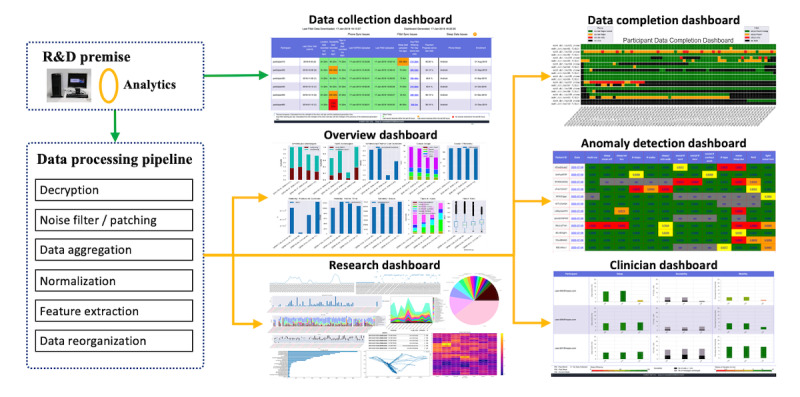
Backend data processing pipelines and dashboards.

For backend R&D analytics, we developed a set of data processing pipelines and various dashboards for monitoring, visualizing, and analyzing data (Figure 2). The data processing pipelines clean (manage missing, duplicated, and erroneous data), convert, and reorganize data into more usable forms. These dashboards are used by research coordinators and clinicians, researchers, data analysts, and technical team members involved in the conduct of the study. A general-purpose research dashboard supports exploratory analytics. In each case, roles and responsibilities determine the access controls for various attributes of the data. Physical controls, supervision, and accountability measures were also deployed to ensure that there was no unauthorized access to data. Further description is given in subsequent sections and more details are provided in the Multimedia Appendix 1.

### Features Supported by the Platform

The following 6 categories of features are obtained from the HOPES smartphone app. In each case, we will indicate *new* if it is a new feature added by us or an enhancement, otherwise, it is an existing feature in the *Beiwe* distribution.

#### Location

GPS coordinates are used to detect deviations from typical travel patterns and to compute a measure of variance or entropy in the locations visited by a participant. To protect user privacy, the raw GPS coordinates are obfuscated via a random displacement (from the origin), which is unique for every participant.

#### Sociability Indices (Some Are New)

In our study, changes in a participant’s s*ociability*, that is, their communication with others, is estimated from available data. Sociability may be reflected in their activities in various forms of messaging and voice/video communications. The original *Beiwe* app can capture incoming and outgoing phone calls and SMS messages. However, in many countries, most people use free social messaging apps as their primary method for text and voice communication; for example, in Singapore WhatsApp use is dominant. We therefore made use of the Android Accessibility Service Application Programming Interface (API) to acquire message metadata from social messaging apps. So far, we have only implemented this for WhatsApp, but it can be easily extended to other social messaging apps. The duration and timing of mobile service phone calls and WhatsApp calls made and received were recorded. Similarly, the length and timing of SMS and WhatsApp messages sent and received were also recorded. Importantly, for privacy protection, we never record or transmit any content of any communication, and we hash the identity or contact number of the counterparty.

#### Finger Taps (New)

Taps provide two types of information that may be related to a person’s health. The speed at which a person taps may give a hint of their neuropsychological function [33]; for example, a fatigued person may tap more slowly or some diseases may cause small, uncontrollable movements. There is also some evidence that finger taps may be used to detect depression [34]. The apps a person uses (determined from their taps) may also give an indication of their status and behavior. For example, a patient with a mental illness who is relapsing might be found to have significantly altered communications, reflected in the number and speed of taps made in the various apps. We sought to capture typing error rates that could be affected by physical or mental conditions. We can determine this from how often the delete or backspace key on the keyboard is tapped. To measure tapping speed, we also need to know whether the person is typing on the keyboard or navigating in a social messaging app. The characteristics and metadata of finger taps on the phone screen were recorded, such as the number and timestamps of taps into apps, different key strokes (from the enter key, delete key, backspace key, alphabet keys, number keys, and punctuation keys), and the group categorization of the tapped apps are also recorded. As a privacy-preservation measure, captured keystrokes are converted into a type token (such as *alphabetic*, *numerical*, and *punctuation*). The app only stores and downloads the type token, and the specific keys that are struck are not recorded.

#### Motion Information (Some Are New)

Accelerometer, gyroscope, magnetometer (new), and pedometer (new) data are recorded to check whether the phone is being moved or is motionless. This information can help determine the amount of phone use and can be correlated with other data collected by wearable devices (sleep, activity, etc).

#### Phone States

The app can record the Wi-Fi state, the Bluetooth state, and the power state (screen on/off and power-down event) of the phone. The Wi-Fi and Bluetooth scan results can, to some extent, provide information about the location of the device, especially when the GPS location is not available. However, these data are sensitive and need to be deidentified and encrypted. The power state feature is usually combined with other features, such as taps, to determine the usage behavior of the phone by the participant.

#### Ambient Light (New)

The app can record the intensity of ambient light through the smartphone’s built-in light sensor (not the camera). This could detect, for example, whether a participant goes out or sleeps in a comfortable sleeping environment; studies have suggested a correlation between a patient’s mental health and their preferred environmental lighting [35]. As sleep and heart rate are important indicators of mental health status, we recorded the following 3 categories of features from the Fitbit wearable (obtained directly from the Fitbit cloud).

##### Sleep

Sleep information during the day and night was recorded, including a breakdown of different sleep stages with time stamps.

##### Steps

The total number of steps in time intervals specified by Fitbit.

##### Heart Rate

The number of heartbeats in time intervals specified by Fitbit. Approximations of other measures of interest, such as heart rate variability, can be computed from heart rate data.

For the HOPE-S study, we captured the following features: location, sociability indices, finger taps, accelerometer, power state, ambient light, sleep, steps, and heart rate.

### Backend Data Processing Pipeline

We have rebuilt the *BBS* backend in Python 3 to systematically process data files, reformat the raw data, and extract high-level features. A considerable amount of feature engineering is being performed on the backend to clean the data, correct data shortcomings, combine different data sources into joint features, and feed various downstream machine learning systems. For example, upon consultation with our clinical partners, we constructed high-level features that are likely to provide useful signals regarding the mental health of the participants in the HOPE-S study. Our current analyses in the study make use of time series of daily or hourly samples of intuitively identified measurements from sleep, steps, heart rate, location, and sociability indices. Some examples include daily totals of the number of hours of sleep, steps, and communications initiated and received. Constructing such features is often necessary in situations with small amounts of or noisy data. For example, when no sleep data are recorded by the Fitbit for a whole day, it is not clear whether the participant did not sleep or whether they just did not wear the Fitbit to bed. We can resolve this ambiguity by looking at the heart rate measurements, which are recorded continually while the Fitbit is worn. If heart rate data are missing for more than an allotted allowance, we can reasonably assume that the participant was not wearing the Fitbit during sleep. As another example, we have developed an Android app grouper that uses information from the Google Play Store to classify all apps into 7 classes defined by us: social messenger, social media, entertainment, map navigation, utility tools, games, and Android systems (other vendor-specific or system apps that cannot be found in the Play Store). This class information is used in the taps data features when classifying a user’s phone activity (eg, *in social media apps*, *in gaming apps*, etc). In summary, this step bridges the gap between data collection and common downstream machine learning modules. Details on the data processing pipeline, high-level feature extraction, and the seven classes of the app grouper are provided in the Multimedia Appendix 1.

We note that although we endeavor to correct *ambiguities* in the data collected by the platform (such as in the example above clarifying truly sleepless nights), we do not make efforts to *impute* missing data. Imputation is required for certain analyses, such as those involving GPS measurements [36]. However, it should be noted that the best imputation method depends on the goals of a particular study.

We are also aware that features that are provided by device manufacturers, such as the pedometer, heart rate, and sleep, are derived using proprietary algorithms that are likely to change over time and are not standardized, nor typically scientifically validated. These features may contain biases or inaccuracies that can affect subsequently trained statistical models. Therefore, our existing data processing pipeline is designed to be flexible enough for researchers to insert on-demand additional steps for data normalization and regularization.

## Platform Improvements

We have made many improvements to the Android app and are in the process of extending these improvements to the iOS app. In this section, we will only describe the most significant improvements; other improvements are provided in the Multimedia Appendix 1. We also used two system variations: the *prototype* or development system and the *deployed* system. Some features may be applied to only one of the systems.

### Scanning Quick Response Codes for Simple User Registration

To facilitate the user registration process and to allow one-way encryption for better data security, study participant kits were prepared and a single-page onboarding document was generated with all the information necessary to onboard a participant. The process was designed for a nontechnical self-service onboarding process. Multiple Quick Response (QR) codes were scanned in the deployed system. They include information on certificate-based authentication to further strengthen security via host verification. The *Additional App Enhancement* Section of the Multimedia Appendix 1 provides details on QR registration.

### Data Compression

To scale the system up to a very large number of users, we need to reduce the utilized communication bandwidth as much as possible. We have therefore added an option when creating a *study* to compress the data before sending it to the server, which may be selected on the backend console by checking the *enable compression* checkbox. Note that data compression is applied before data encryption. This feature was only implemented in *the prototype system*.

### Security Enhancements

The HOPES platform is redesigned on top of *Beiwe* to ensure data confidentiality, data integrity, and high availability, and to enable system auditing and user authentication. The design also supports large-scale deployments with a distributed pipeline. Finally, the design emphasizes a separation of these duties throughout the architecture to minimize the risk of data breaches and to preserve data privacy throughout the lifecycle of a study. In the original *Beiwe* platform, data are decrypted in the data collection server and re-encrypted using the study key. This poses a certain amount of risk because the data collection server directly faces the public internet. In our HOPES platform, data are encrypted at all times while on the phone and in the data collection infrastructure and are only decrypted in clinical or R&D premises. The decryption key is only accessible from clinical or R&D premises; therefore, in principle, the data are not decryptable on the phone or in the data collection infrastructure. Data are only reidentified when needed for qualified clinical purposes and only by clinical staff.

## Dashboards, User Interfaces, and Data Analysis

**Figure 3:**
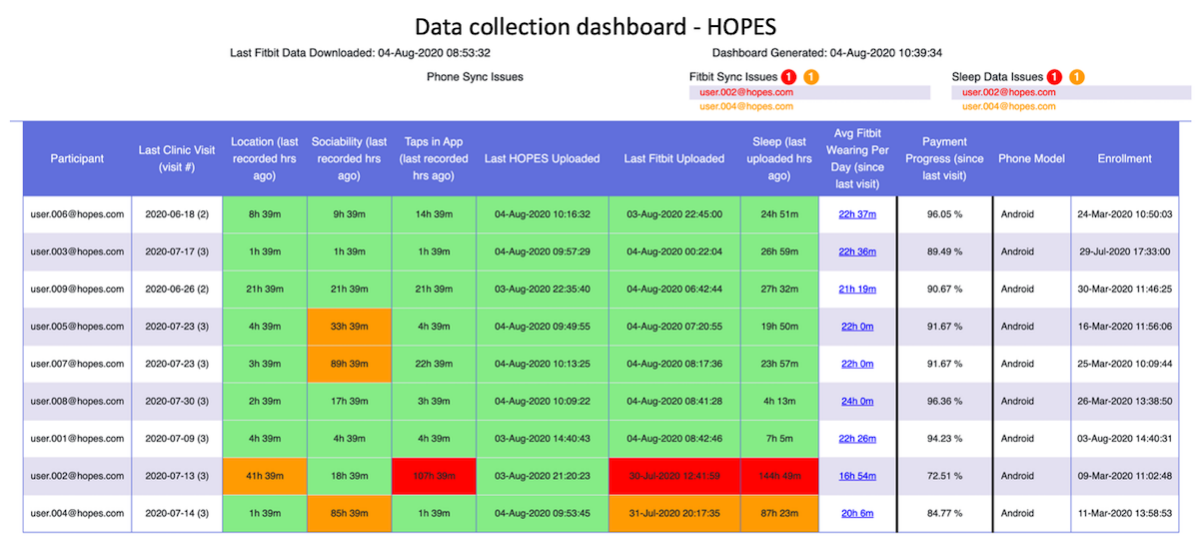
The data collection dashboard shows the data uploading status of all participants.

Ensuring complete data collection is important. A variety of issues can result in not receiving data as expected, including technical failures, participants not adhering to the guidelines on device usage, or participants failing to wear their device. Monitoring this process is particularly challenging at scale. Therefore, we created a *data collection dashboard* (Figure 3) to facilitate the monitoring of the data collected.

*The data collection dashboard* is populated using the metadata extracted during the downloading phase of the Fitbit and phone data. The AWS Lambda function (which is set to trigger every 5 minutes) is set up to retrieve these data from their respective S3 buckets and create an HTML file. To fill the dashboard to ensure that the participants comply with the study requirements, the following data types are observed and closely monitored: location, sociability, taps in app, last HOPES upload, last Fitbit upload, and sleep. Color codes denote the data collection status: red meaning need to take an action, orange meaning need to closely monitor, and green meaning normal.

The data collection dashboard does not require decrypted data and is thus constructed before decryption. As a result, it can be hosted on an upload server with little security risk. However, it does not show the full historical data completion status, which is sometimes needed. Hence, we developed the *data completion dashboard*, which is described in detail in the Multimedia Appendix 1.

### Data Visualization Toolkit

We developed a *data visualization toolkit* to visualize and explore the collected data. The toolkit can also perform some basic statistical analyses, such as the comparison of features between defined date ranges. For further details on the usage and capability of the data visualizer, see the Multimedia Appendix 1.

### Clinician Dashboard

**Figure 4:**
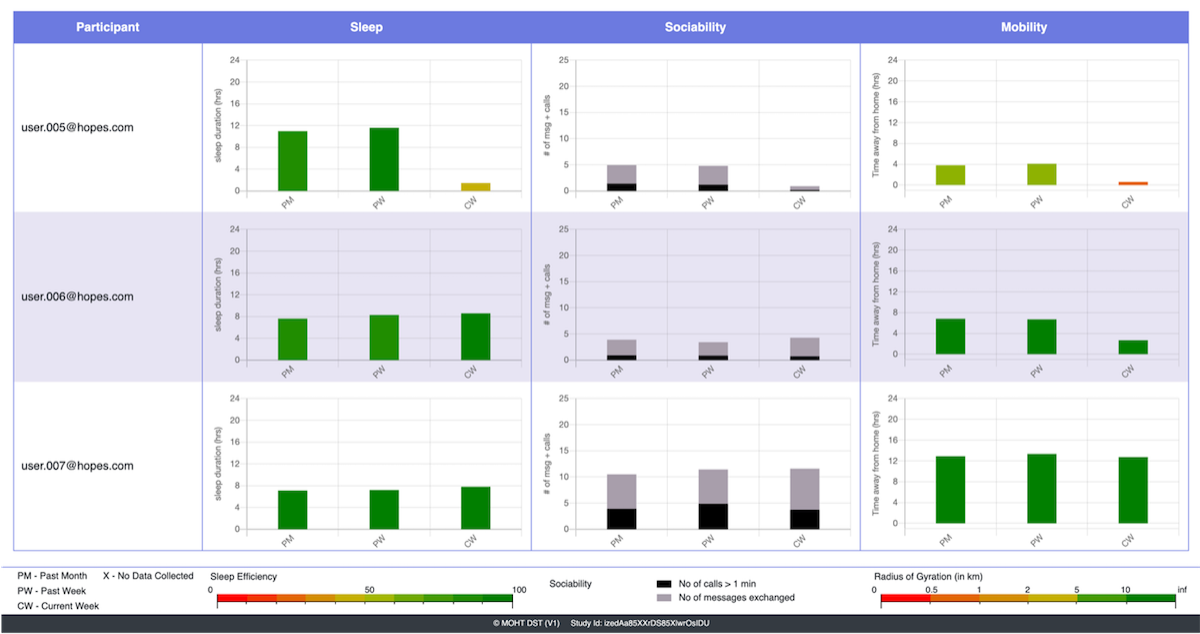
The clinician dashboard shows a preview of general trends in patient biomarker data.

The *clinician dashboard*, illustrated in Figure 4, is designed for clinicians to preview general trends in participants’ digital phenotyping data and may be useful during clinical encounters. On the basis of previous studies and the observations of our clinical partners, we decided to report sleep, sociability, and mobility data for the current version of the clinician dashboard.

*Sleep* is plotted based on total sleep duration and sleep efficiency; the latter is depicted by color. Sociability is plotted using the number of messages exchanged and the number of calls for a duration of more than 1 minute. Mobility is based on the time away from home (time spent away from sleeping location) and the radius of gyration (maximum distance traveled from home). These graphs are drawn based on averages over 3 timeframes: the current week is 7 days before 0:00 AM of the current day, the past week is 7 days before the current week, and the past month is 30 days before the past week. An example further explaining the clinician dashboard can be found in the Multimedia Appendix 1.

### Anomaly Detection Dashboard

To support a wide variety of applications attempting to analyze and identify interesting changes among the many features being collected by the platform, we implemented a generic purpose *anomaly detection system and dashboard*. The system comprises several anomaly detection algorithms on the backend that report their findings via a dashboard. The dashboard is designed to create alerts about possible irregularities arising in the digital phenotyping data each day.

There are many machine learning approaches to anomaly detection in time-series data. One approach is to train a time-series model on historical data and compare new data with forecasts from this model, *scoring* the predictions based on how *good* or *bad* they are. For example, a simple scoring mechanism compares the empirical distribution of the *residuals* (ie, the errors of the fitted model’s predictions on the training set) to the realized prediction error on new data.

We have experimented with several time-series models, including the broad class of autoregressive integrated moving average models [37] and the class of *Gaussian processes* [38], fitting them to a subset of digital phenotyping features that were initially selected as important for our HOPE-S study (see the Multimedia Appendix 1 for details of the features). We note that these two choices of models are able to capture *periodic effects*, which are important for our HOPE-S study, as participants’ behaviors may change markedly on the weekends. Selecting the most appropriate model depends on the data and the application at hand. We train the models every day on all past data and compute the predictions of the digital phenotyping features for the next day. At the end of the following day, the realized digital phenotyping features were compared with the predictions and scored, and these scores were transformed to be interpreted as *the probability that the observed data is an anomaly*. Therefore, the final score is a number between zero and one, where higher values constitute alerts.

**Figure 5:**
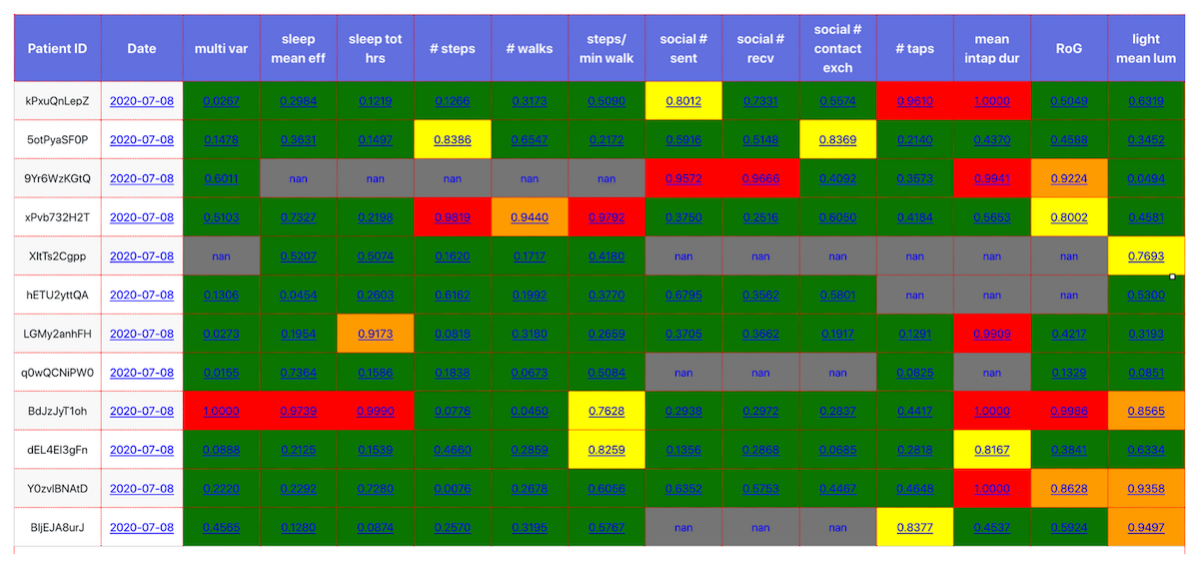
The anomaly detection dashboard with a visualization of the scores from a collection of anomaly detection models.

In Figure 5, we display an example of what the anomaly detection dashboard looks like on a given day. Each row corresponds to a participant, and each column corresponds to a different anomaly detection score. The participant’s identifier and the last date their scores were successfully updated are displayed, along with the anomaly scores for each feature. The score from a multivariate model is also displayed, which may capture interdependencies between features that affect whether a measurement is anomalous. For example, major disruptions in sleep naturally coincide with periods of long-distance travel (a large radius of gyration). Note that the cells are colored according to the severity of the scores.

Although this dashboard is mainly used for research at this point, if reliable anomalies are detected, they can be promoted to the clinician dashboard. In the context of our HOPE-S study, it was shown that digital phenotyping signals from patients with schizophrenia exhibit a measurable increase in anomalies in the period leading up to a relapse event [11]. However, as an unsupervised learning problem, the performance of an anomaly detection routine is dependent on the context of its application, and users will likely have to adjust the underlying algorithm to suit their needs. Therefore, we made the dashboard modular, where the anomaly detection routine can be replaced on the backend with customized routines without affecting its exposure to the user-facing dashboard.

## Example Analyses

### Data Completion Rate Analysis

The completeness of the data collected in the HOPE-S study depends on the technical stability of the platform and participant compliance. There will always be situations that are difficult to anticipate and can cause losses of data (eg, a few participants had their Fitbit wrist bands broken in the middle of the study, and while waiting for the replacement band to arrive, they were unable to wear the device).

Table 1 shows the data completion rates for each low-level feature. The rate is computed as the number of days with feature data divided by the number of days enrolled in the study. For phone features such as call log and SMS log, which can have no data if the participant really has no call/SMS during that day, we checked the presence of empty, time stamped feature files stored by the platform to determine whether that feature is being successfully collected. Our researchers and clinical partners generally felt that the overall completion rate was satisfactory.

**Figure 6:**
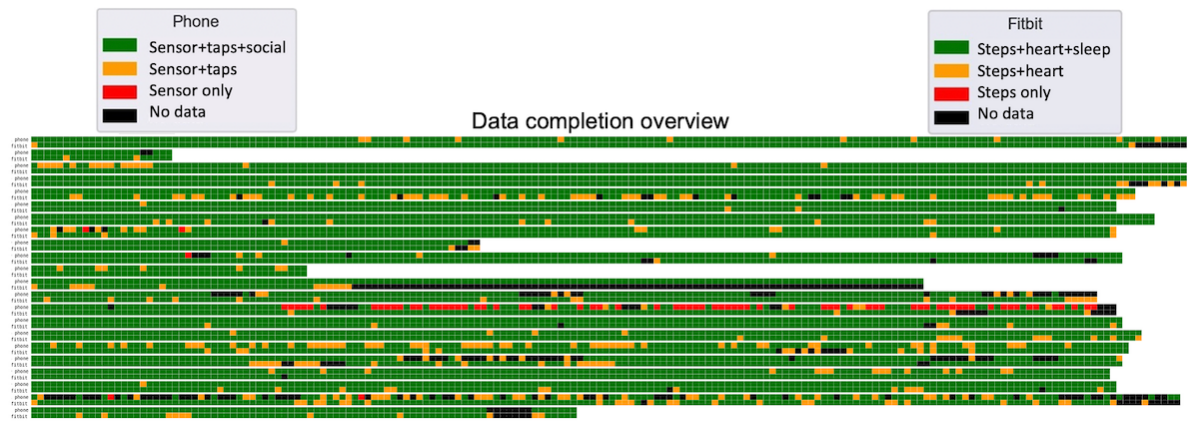
Data completion overview for the first 22 participants who have already completed the study (odd rows refer to phone data; even rows refer to Fitbit data).

Figure 6 provides a dynamic, graphical overview of the data completeness for each participant. Each participant had 2 rows in the display: the first for their phone features and the second for their wrist device features. Each square represents the completion status for a single day (refer to the legend for information on color coding). This dashboard has proven valuable to our researchers and clinical partners when following up with study participants to improve compliance and to quickly resolve any technical issues that may arise.

**Table 1:** Data completion rate on 22 participants that have completed the study.

Raw feature name	Data completion rate (%)
Accel	87.0
Call log	94.6
Power state	94.3
Sociability call log	94.6
Accessibility log	87.2
GPS	93.0
Sleep	87.1
Sociability msg log	94.6
Ambient light	91.3
Heart	93.5
SMS log	94.6
Steps	96.9
Taps log	89.7
Overall	92.2

### Example of Use: Measuring the Effect of Singapore’s Circuit Breaker

In response to the SARS-CoV-2 (COVID-19) pandemic, Singapore imposed a stay-at-home order or *cordon sanitaire*, which is formally called *the 2020 Singapore Circuit Breaker measures* or CB. This lockdown was in effect from April 7, 2020 to June 1, 2020, after which gradual stages of reopening have occurred. During this period, people were required to stay at home as much as possible, avoid nonessential travel and social visits, and maintain social distancing in public. We expect the lockdown to have an effect on some digital phenotyping features. As a test for our digital phenotyping system, we performed and reported a data comparison using 22 participants’ data before and after the start of this *CB*.

Table 2 shows a subset of features that show statistically significant differences before and after the CB began on April 7, 2020. Not surprisingly, as people were required to stay at home, the time at home has increased, and the number of significant locations visited has decreased. Features related to physical activity (heart rate, steps, and acceleration) also decreased, as might be expected. Both sleep and sleep efficiency decreased among these participants. It is also noteworthy that participants appear to use a fewer number of apps, perhaps because there is no need for some apps such as maps for navigation or those checking bus arrival times; however, it appears that they spend more time in entertainment apps. Moreover, the ambient light indoors is generally dimmer than it is outdoors, therefore, the observed decrease in maximum ambient light is also as expected.

**Table 2:** Comparison of 6 weeks of digital phenotyping data before (from 45 days before to 3 days before) and after (from 3 days after to 45 days after) the start of Singapore’s Circuit Breaker on April 7, 2020.

Feature name		6-week before CB^a^ starts, mean (SD)	6-week after CB starts, mean (SD)	*P* value (paired *t* test)	*P* value (Wilcoxon signed rank test)
**Smartphone features**					
	accel_L_std (L: length of the accel. vector)	0.526 (0.325)	0.370 (0.345)	<.001	<.001
	accel_ddt_max (ddt: time derivative)	0.00807 (0.0044)	0.00551 (0.0041)	.005	<.001
	ambientLight.hourly_max_log1p_lux	2.538 (0.721)	2.190 (0.587)	.001	.001
	callLog_Incoming Call	0.533 (0.835)	0.288 (0.560)	.001	<.001
	gps-mobility_Hometime / mins	1111 (292)	1328 (143)	<.001	<.001
	gps-mobility_SigLocsVisited	1.354 (0.429)	1.220 (0.288)	.01	.02
	powerState.hourly_n_screen_on	6.716 (2.77)	5.180 (2.44)	.008	<.01
	tapsLog.daily_n_unique_apps	16.10 (4.54)	14.24 (4.47)	.04	.04
	tapsLog.daily_n_taps_in_entertainment	293.9 (259)	378.3 (365)	.07	.05
**Wrist-wearable features**					
	steps.daily_n_steps	3921 (2435)	2400 (1807)	.001	<.001
	steps.daily_n_mins_walk	113.8 (53.23)	79.78 (54.24)	<.001	<.001
	heart.daily_HR_mean	82.89 (10.48)	80.35 (9.04)	.10	.03
	heart.daily_HR_min	56.08 (6.85)	55.74 (7.23)	.77	.26
	sleep_total_hrs	9.196 (1.94)	8.727 (1.98)	.31	.096
	sleep_mean_efficiency	93.27 (3.08)	92.39 (3.38)	.04	.009

^a^CB: circuit breaker.

We compared our results with another study based on Fitbit use: the Health Insights Singapore (hiSG) study [39]. The daily step count decreased by approximately 35% in our study and approximately 42% in hiSG; the minimum heart rate decreased by 1.1 bpm in our study, and the resting heart rate decreased by 1.6 bpm in the hiSG study; sleep efficiency decreased by 0.8% in our study and by 0.2% in the hiSG study. All comparisons between both studies were consistent in demonstrating changes before and after the onset of the CB measures in Singapore.

### Conclusions and Future Work

Digital phenotyping is a promising area in health care, but great care and effort is required in designing a system that is easy to use, is safe in terms of data security and privacy, and collects data with sufficient detail and reliability to be useful in research and patient care. We found the *Beiwe* platform to be a suitable base that can be used and extended to create the HOPES platform. Our main extensions have been adding many more sources for data collection, integrating the use of a wearable device, and the development of a large set of participant monitoring and management platforms.

We were also driven by the requirements of a clinical research study for schizophrenia (HOPE-S). This required us to develop significant enhancements in security, privacy, ease of use, and scalability, choosing a careful combination of public cloud and on-premises operation.

We needed to create new mechanisms to clean, process, present, explore, and analyze the massive and diverse data collected when digital phenotyping. These need to serve the needs of clinical research study operations, clinical care, platform developers, and researchers, and hence a range of data pipelines and tools for data analysis have been developed.

Our initial platform is in use in the HOPE-S clinical trial, and interim results will soon be reported. An initial analysis using SARS-CoV-2 as a test case yielded meaningful and expected results consistent with expected lockdown behaviors and was consistent with an independently conducted study in the same country.

Currently, we are considering making the HOPES platform open source for the research community to access. We continue to add features and make adjustments for newer versions of the Android operating system. Simultaneously, we are working on an iOS version of the app to access a wider range of users with Apple devices.

## Appendix

Multimedia Appendix 1 Further technical documentation on the solution architecture, App enhancements, added digital phenotyping sensors and features, the system’s Fitbit component, the back-end data processing pipeline, and the dashboards.

## Abbreviations

API: Application Programming Interface
AWS: Amazon Web Services
CB: circuit breaker
HOPES: Health Outcomes through Positive Engagement and Self-Empowerment
HOPE-S: Health Outcomes via Positive Engagement in Schizophrenia
iOS: iPhone Operating System
QR: Quick Response
R&D: Research and Development
