# Supplementary material for: HOPES: An Integrative Digital Phenotyping Platform for Data Collection, Monitoring, and Machine Learning
Source: J Med Internet Res. 2021 Mar 15;23(3):e23984. doi: 10.2196/23984 (PMC8074871; doi:10.2196/23984)
Supplement: Multimedia Appendix 1 [file jmir_v23i3e23984_app1.docx]

Supplementary Material: HOPES - An Integrative Digital Phenotyping Platform for Data Collection, Monitoring and Machine Learning

Xuancong Wang^1^, Nikola Vouk^1^, Creighton Heaukulani^1^, Thisum Buddhika^1^, Wijaya Martanto^1^, Jimmy Lee^2,4^, Robert JT Morris^1,3^

^1^Ministry of Health Office for Healthcare Transformation (MOHT), Singapore

^2^Institute of Mental Health, Singapore

^3^National University of Singapore, Singapore

^4^Lee Kong Chian School of Medicine, Nanyang Technological University, Singapore

Corresponding Author: Creighton Heaukulani ([creighton.heaukulani@moht.com.sg](mailto:creighton.heaukulani@moht.com.sg))

Abstract

This paper provides supplementary material for our main paper (titled “HOPES - An Integrative Digital Phenotyping Platform for Data Collection, Monitoring and Machine Learning”) in which we describe the development of, and early experiences with, a comprehensive Digital Phenotyping platform: **H**ealth **O**utcomes through **P**ositive **E**ngagement and **S**elf-Empowerment (HOPES). HOPES is based on the open-source *Beiwe* platform but adds a much wider range of data collection, including the integration of wearable devices and further sensor collection from the smartphone. Requirements were in part derived from a concurrent clinical trial for schizophrenia. This trial required development of significant capabilities in HOPES for security, privacy, ease-of-use and scalability, based on a careful combination of public cloud and on-premises operation. We describe new data pipelines to clean, process, present and analyze data. This includes a set of dashboards customized to the needs of research study operations, and for clinical care. A test use case for HOPES is described by analyzing the digital behavior of 20 participants during the SARS-CoV-2 pandemic.

1. **HOPES Solution Architecture**

The HOPES platform infrastructure was separated out into functions of administration, data upload, data encryption, wearable data collection, operational management. Figure 1 shows the separation of the layers between the cloud environment and the on-premise data analytics environment. The data collection, monitoring, and aggregation infrastructure are separated into logical networks that share common encrypted storage, and access to appropriate encryption keys to ensure the data collected is consistent. Access to the encrypted study data, and the study decryption keys is exclusively provided to authorized data analytical services on the R&D Premise for processing. The collection of wearable data from cloud data collection sources is independent of the collection of HOPES application data, but both are normalized into a common set of formats and encrypted using the public key of each respective participant’s data to ensure consistent data processing. The private key is only accessible to authorized data analytics pipelines.

Figure 1: HOPES Multi-Tier Scalable Distributed Architecture

The solution has separated administration to only authorized administrators accessing through private VPN connections. Monitoring dashboards are separated into a different logical network accessible through a different VPN gateway, and only metadata is visible to operators. Secure download is made possible through private authorized connections, and secured credentials. Once data is processed by the analytics pipeline, de-identified processed data is made available to data scientists and clinicians for further analysis visible in the data exploration tooling, anomaly dashboard, clinician dashboard, and as raw information for further analysis. Data is encrypted at all times while in the data collection infrastructure, and only decrypted during the analytics pipeline.

Figure 2 Scalable Infrastructure

Figure 2 represents the data collection infrastructure enhanced to leverage the best cloud and security architecture through separation of capability from data collection, data administration, as well as auto-scale and load balancing at every level. The infrastructure is stateless transaction-to-transaction. The architecture implements a secure certificate-based authentication and a rotating credential to ensure only authorized valid participants are connecting into the infrastructure. Automated patching, web application firewalls, distributed-denial of service protection, credential vaults, and secure software development practices ensure a robust integrity for the infrastructure. An automation framework was written to deploy the software reliably and securely.

1. **Additional App Enhancement**

**2.1 QR Scanning for Participant Registration**

To facilitate user registration, we implemented a QR code reader to replace manual entry of credentials. To register a participant’s phone to the study server, the registration information is generated on the server side with an asymmetric encryption and decryption key. The registration information together with the encryption key is stored in the QR code and can be sent to the participant via email or as a print-out. A migration feature was added to allow for users to migrate to new study phones and maintain the de-identification credentials as well as maintain integrity of study data.

| 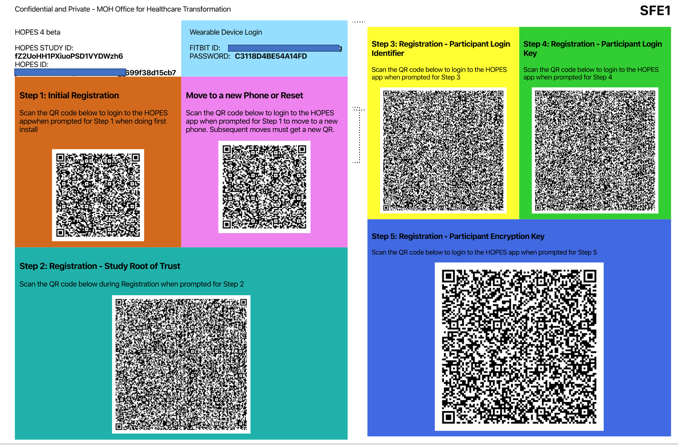  Figure 3a: Study Onboarding Document | 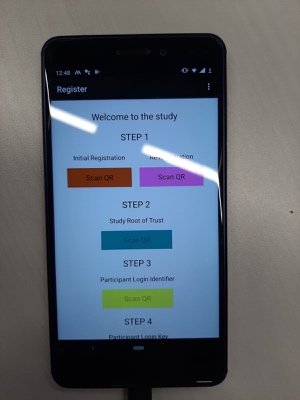  Figure 3b: HOPES Onboarding | 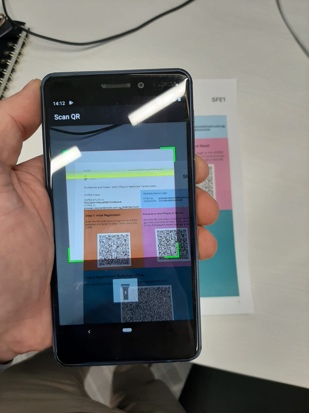  Figure 3c: QR Scan | 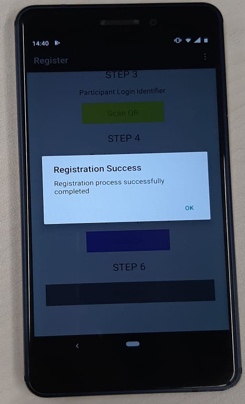  Figure 3d: Registration Complete |
| --- | --- | --- | --- |

As shown in Figure 3(a-d), the participant’s onboarding sheet is securely generated by the study administrators, passed to the clinicians for onboarding, and then when participants are ready to be onboarded, the sheet is used to configure the HOPES app by scanning the QR codes. The onboarding consists of six color-coded steps used to input the necessary information for logging into the server, defining the data randomization, and connecting into the infrastructure. For simplicity, and the application ensures each step is followed in order. The QR codes contain the randomized de-identification information, the encryption keys for encoding the data, as well as secret key to connect into the infrastructure. In the latest version, the QR codes are encrypted to ensure confidentiality of the information offline. The keys are only valid during the study duration, and are invalidated upon participant completion of the study, or the study completes. This information is never made available during processing, and securely stored offline by the study administrators, and destroyed after the study is complete.

This process has allowed us to onboard participants easily without the error of inputting credentials, server addresses, facilitate self-service onboarding, and simple onboarding for our participants.

### **2.2 HOPES Debug Interface**

Figure 4: the HOPES Debug Interface


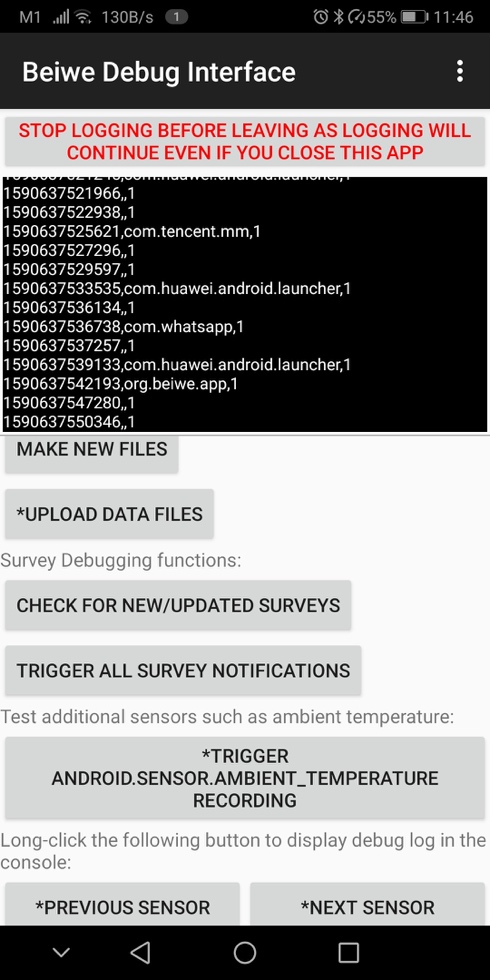


**HOPES Debug Interface**

The debug console can help test almost every functionality of the app directly on the phone without the need to connect to a computer running Android Studio. It logs every feature that can be collected. It serves two main purposes: for technical troubleshooting (certain features may work differently on certain brands of phones with certain Android versions) and to give more technically-oriented users or inquirers some degree of privacy assurance by showing what exact information is collected and sent to the server. For example, Figure 4 shows an example of the taps log being captured. The debug console can connect to every feature logger. Data from that feature’s listener can be displayed in the console before being encrypted and written to file. The debug console is directly accessible when no study has been registered. After study registration, it will be locked by a password or totally disabled depending on the study settings. This is to prevent interference to the data collection for the study.

1. **Newly-Added Digital Phenotyping Features**

Below, we describe features that are newly added or enhanced on top of the Beiwe distribution, expanding on those already stated in the main paper.

- - 1. Sensor: Pedometer

Although step counts are readily captured by most wrist-wearable devices like Fitbit, it is still beneficial to capture step counts on the phone, since some people do not wear smart bands or watches. For those that do wear wrist devices, the differences between the two sources of step data can provide some interesting information. For example, if the wrist device registers steps during a particular period but phone does not, this may suggest that the user is merely walking around their home or office (i.e., not travelling), in which case their phone may have been left on a desk or table.

- - 1. Sensor: Ambient Light

We capture ambient light since studies have suggested there is correlation between a patient’s mental health and their preferred environmental lighting. Additionally, the ambient light in a person’s sleep environment would likely affect sleep quality, which may in turn have an influence on their mental wellness. In our implementation of the “study settings”, the researcher can set a time interval during which the ambient light reading is taken.

- - 1. Sensor: Magnetometer

The magnetometer returns the direction of magnetic fields passing through the phone. This information tells us about the orientation and alignment of the phone, which in turn can determine the rotational motion of the phone.

- - 1. Capturing Taps

Taps provide two types of information that may be related to a person’s health. The speed at which a person taps may give a hint of their wellbeing; for example, a fatigued person may tap more slowly, or some diseases may cause small, uncontrollable movements. The apps a person uses (determined from their taps) also gives an indication of their behavior. For example, a relapsing schizophrenia patient may have significantly altered communication, reflected in the number and speed of taps he/she made in each app. We make use of Android application overlay to capture taps. In particular, it creates an invisible tiny popup window on the screen and watches for every tap outside the window. It then queries the Android Usage Stat Manager to get the app name in which the last tap was made. We record the timestamp, app-name, and screen orientation of the phone for each tap.

- - 1. Accessibility Taps

The taps-capturing method (described in Section 4.1.4) using the Android application overlay cannot capture Android system buttons such as Home, Back, and Recent Apps. Additionally, it cannot capture more detailed information about the button that has been tapped due to Android’s privacy preserving strategies. We attempt to capture typing error rates, which we believe can be affected by a person’s the physical or mental condition. We can determine this from how often the DELETE key on the keyboard is tapped. To measure tapping speed, we also need to know whether the person is typing on the keyboard or navigating in a social messaging app.

- - 1. Sociability Messages

For our study, changes in a participant’s sociability (i.e., their communication with others) may be related to his/her mental health status. This may be reflected in their activity in social messaging apps. In particular, the number of incoming and outgoing messages, the number of message senders to which the participant has replied, and the lengths of outgoing messages, for example, may indicate a degree of social engagement. The original Beiwe app captures incoming and outgoing SMS messages. However, in Singapore most people use social messaging apps like WhatsApp as their primary method for text communication. We therefore make use of the Android Accessibility service privilege to acquire message meta data from social messaging apps. We have so far only implemented this for WhatsApp, but it can be easily extended to other social messaging apps in the future. To protect user privacy, we do not capture the content of message, but only the meta data of each message, i.e., the timestamp, direction (incoming or outgoing), sender/receiver hashed identity (not their names), message length, and message type (image, text, voice, etc.).

- - 1. Sociability Calls

Similar to Sociability Messages, our app also leverages the Android Accessibility service privilege to capture calls within social messaging apps since, in Singapore, a significant number of phone calls are made using social messaging apps rather than the phone’s SIM card. Following the data format of the call log feature implemented in Beiwe, the HOPES app records the timestamp, duration, direction (incoming or outgoing), type (voice or video call), and hashed sender/receiver identity of every call within WhatsApp. Depending on the need, this can be easily extended to other social messaging apps.

- - 1. Time Zones

In general, changes in time zone can have a significant impact on both one’s physical and mental health, as well as one’s usage data. If not handled properly, it can cause large discrepancies in clinical predictions for participants who often travel across time zones. For every feature that we capture, we have therefore added one additional field recording the current system time zone.

1. **Additional Information on HOPES Fitbit Component**

## **Fitbit Setup**

We used the Fitbit Charge 3 in our study. User accounts were pre-created with system generated email addresses and passwords. Separate “main” application was created in order to download data from user accounts, following “Authorization Code Grant Flow”. Data was downloaded using the Fitbit Web API, in terms of intraday time series.

## **Fitbit Data Download Architecture**

We used *AWS lambda*, to extract user data from the Fitbit cloud, update access tokens, write encrypted data to files, calculate meta data, and then upload into *AWS S3 buckets*. The Fitbit data downloader lambda is triggered every hour by a rule setup in *AWS CloudWatch.* Access token of each user was setup in the *AWS Secret Manager* and updated as necessary.

1. **Back-end Data Processing Pipeline**

The data processing back-end is designed to reformat and process data for downstream machine learning models. We created a master script called *./periodic-run.sh* which is scheduled to run periodically with a configurable time interval. Sequentially, it will:

1. Decrypt new data files (output to *./decrypted*). In the original system design, for every participant, for every feature, one file will be generated every hour. The file is uploaded and stored on server in encrypted form. The backend data processing server keeps all of these files in both encrypted and decrypted format (for decryption troubleshooting). The script will recursively decrypt every file in the directory if their decrypted version does not exist.
2. Patch new data files (output to *./1.decrypted*). This will fix some file format inconsistency issues, e.g., the timestamp in some device feature files are in units of seconds rather than the milliseconds used in phone features.
3. Concatenate all files (output to *./2.decrypted*) for the same participant and feature category, fix corrupted files, ignore duplicate files, etc.
4. Fix some data value issues (output to *./3.decrypted*), such as clamping the ambient light value range (on certain phones, the ambient light sensor return lux values like 1e+15 upon saturation, while the authentic maximum value is around 65k), fix duplicate and corrupted data entries, and add the app-group column, see Section 4.3.2 for details). As compared to Step 2, this steps fixes issues after file concatenation.
5. Summarize into high-level features (output to *./4.decrypted*). The raw data comes as irregularly-spaced time-series data. We use conventional methodology to extract high-level regular time-series features from this raw data. We typically extract summary statistics such as the maximum, minimum, mean, median, standard deviation, count, etc., of some features over at an hourly or daily sampling interval. For the detailed specification, see Table 1 and Section 5.1.
6. Concatenate all features and combine all data files (output to *./5.decrypted*). Some of the features are hourly features and some are daily features. For hourly features, we concatenate the vectors from all hours in one day to form a feature for that day. Next, the system first combines every participant’s daily feature data into one compressed file, and it then combines all participants’’ data into a single (rather large), compressed file.

| Feature Name | Source | # of dims. daily | # of dims. hourly | Total Dimensions |
| --- | --- | --- | --- | --- |
| Sleep | Fitbit watch | 17 | 0 | 17 |
| Steps | Fitbit watch | 14 | 4 | 110 |
| Heart rate | Fitbit watch | 12 | 6 | 156 |
| GPS mobility | smartphone | 15 | 0 | 15 |
| Accelerometer | smartphone | 0 | 5 | 120 |
| Accessibility taps | smartphone | 0 | 1 | 24 |
| Call logs | smartphone | 5 | 0 | 5 |
| Ambient Light | smartphone | 3 | 2 | 51 |
| Power state | smartphone | 6 | 2 | 54 |
| Sociability call log | smartphone | 4 | 0 | 4 |
| Sociability msg log | smartphone | 9 | 0 | 9 |
| Taps | smartphone | 13 | 6 | 157 |
| SMS | smartphone | 7 | 0 | 7 |
| Total | (both) | 105 | 26 | 729 |

Table 1: Summary of all the extracted and processed high-level regular time-series features (dims, or dimensions) from raw digital phenotyping features

Our design of the data processing pipeline caters to contemporary machine learning (ML) models. Depending on the model and the scale of the study, any stage of output from *./3.decrypted* to *./5.decrypted* can be directly plugged into any downstream analysis. For example, in Step 5, there will be information loss due to feature summarization, so some ML models may preferably use a 2-stage or multi-stage recurrent neural network (RNN), in which the first-stage RNN reads the raw event time-series data, one RNN for each kind of feature, followed by subsequent RNNs combining the output of all those first-stage RNNs to predict an output. Such a model would take its inputs from *./3.decrypted*.

- 1. **Specification for High-level Feature Extraction**

The purpose of this specification is to convert the raw data into regular daily time-series data, i.e., one fixed-dimensional vector every day for every participant, and to ensure value continuity (e.g., sleeping at 23:59 and 00:01 are very close, so should be reflected in the values as well) for downstream machine learning models.

1. Accelerometer (accel.csv)

INPUT: every 10 minutes (or longer), we have 0-3500 triplets of (x, y, z)

OUTPUT: every hour, max/min/std/mean of $\sqrt{x^{2}+y^{2}+z^{2}}$; and max rate of change of acceleration, i.e., $\text{max}\left( \left| \begin{aligned} \Delta x/\Delta t \\ \Delta y/\Delta t \\ \Delta z/\Delta t \end{aligned} \right| \right)$.

1. Accessibility (accessibilityLog.csv)

OUTPUT: every hour, the total number of taps; the number of keyboard taps; the number of DELETE key taps; the ratio of (# of DELETE key taps)/(# of keyboard taps)

1. Call log (callLog.csv)

INPUT: a log of every SIM phone call, with timestamp, incoming/outgoing, hashed identity and call duration

OUTPUT: [same as sociabilityCallLog, wherever applicable]

1. Location (locTime-gps.csv)

[We follow Beiwe-Analysis sample code.]

1. Heart rate (heart.csv)

INPUT: every 5 seconds, the heart-rate (HR) value in beats per minute, (if not wearing, take the previous value)

OUTPUT: every hour, max/min/std/mean of HR, max/min/std of HRV, (i.e., $\Delta\text{HR}/\Delta t$); every day, max/min/std/mean/median of HR, max/min of HRV, mean/std of abs(HRV), 25% and 12.5% quantile of HR (since minimum heart-rate can be quite volatile).

1. Ambient light (light.csv)

INPUT: every 5 minutes (or longer), a value in units of lux ranging from 0 to ~50k

OUTPUT: every hour, max/mean/min of $\text{log}\left( 1+value \right)$, 50-high value (the mean of the top 50% of values)

1. Power state (powerState.csv)

INPUT: a log of every event such as screen turn on/off, power-down signal, device idle state change, etc.

OUTPUT: every hour, the number of seconds while the screen is on and the total number of power events (screen-on, screen-off and power-off); every day, the number of power-down signals, max/min/std/mean duration (in seconds) of each screen-on session

1. Sleep (sleep.csv)

INPUT: every day, during estimated sleep hours, a time log of the type (deep, light, REM and awake) and duration of every sleep segment (with 30s resolution)

OUTPUT: every day (from 15:15 to 15:15 the next day), the total duration of deep/light/rem/awake segments respectively; the number of awake segments inside the main sleep; the number of awake segments $\geq180$ seconds inside the main sleep; the ratio of (total duration of deep/light/REM/awake segments inside main sleep)/(total duration of main sleep) respectively; the starting and ending time of the main sleep with respect to 7:15 and 23:15; time-to-asleep (time from night going to bed to falling asleep) and time-to-getup (time from waking up in the morning to getting out of bed); sleep efficiency.

1. Sociability call log (sociabilityCallLog.csv)

INPUT: a log of every WhatsApp call event

OUTPUT: every day, number of incoming calls; number of outgoing calls; number of missed calls; total duration of calls; number of people talked (either incoming or outgoing, but must have $\text{duration}>0$)

1. Sociability message log (sociabilityLog.csv)

INPUT: a log of every WhatsApp message event

OUTPUT: every day, number of received messages; number of sent messages; total length of received messages; total length of sent messages; number of contacts who has 1) only received message, 2) both received and sent messages, and 3) only sent messages, respectively.

1. Steps (steps.csv)

INPUT: every minute, number of steps made

OUTPUT: every hour, total number of steps, max number of steps in a minute within that hour; every day, total number of steps, # of wearing minutes, # of minutes with steps, # of walks (a walk is at least 3 consecutive minutes with each minute having at least 10 steps), the max/mean steps during each walk, the max/mean duration of each walk, the average steps per minute during each walk, max number of consecutive minutes each with more than 3 steps and 30 steps (to predict whether have gone outdoor).

1. Taps (tapsLog.csv)

INPUT: a log of every tap, with timestamp, screen orientation and in-app name

OUTPUT: every hour, the total number of taps, the max/min/std/mean/median of inter-tap duration while screen is on; every day, the number of distinct in-app names, the number of taps made in each APP category (games, social media, social messenger, etc.), and the max/min/std/mean/median of inter-tap duration in social messenger.

1. SMS log (textsLog.csv)

INPUT: a log of every SMS, with timestamp, send/receive, hashed identity and message length

OUPUT: [same as sociability message log, wherever applicable)

- 1. **Android App Grouper**

A person’s behavior and mental health state is to some extent reflected in the kind of apps they spend their time in. We developed an app-grouper which classifies all apps into 7 groups, according to each app’s package name and Google Play Store’s categorization of the app.

1. **Social Messenger**: apps that involve person-to-person real-time communication, e.g., *WhatsApp, QQ, Wechat, Telegram, Dating apps, Email*, etc.
2. **Social Media**: apps that involve information exchange with the world, e.g., *Youtube, Chrome, Firefox, Facebook, Instagram, Twitter*, etc.
3. **Entertainment**: all apps related to education, comics, stories, sports, music and video, etc., for personal life entertainment but are not classified under games
4. **Map Navigation**: apps that are related to travel, map and navigation, e.g., *SGBus, SG BusLeh, Maps, MAPS.ME*, etc.
5. **Utility Tools**: apps that are used for various utility purposes such as banking, finance, document/photo/video viewer and editors, e.g., *PayLah, PayNow, OCBC, AXS, Dropbox, WPS Office, PDF reader*, etc.
6. **Games**: all apps in the game category of the Play Store
7. **Android System**: all built-in apps that cannot be found in the Play Store, e.g., Phone call, SMS, Camera, Gallery, Contacts, Settings, etc. Take note that for some of the apps, their package names might not be the same across all brands of phones, thus, we decide not to further classify them.
8. **Data Visualization Toolkit**

The data visualization toolkit has been developed as a web interface using *Jupyter notebook*. The dashboard is highly configurable. The user needs to specify an input root path, inside which the directory structure can be either ‘*RootPath/StudyName/PatientName/FeatureName/timestamp-files*’ or ‘*RootPath/StudyName/PatientName/FeatureName.csv(.gz)*’, i.e., it can view data files both before and after concatenation (as described in Section 4.3). In the *master configuration dashboard* (as shown in Figure 5), the user can choose which study, participant, and feature to view. In other checkboxes, drop-down lists, and sliders, the user can set various options and choose different types of graphs to plot. For example, given the heart-rate data (which is in the form of a time-series with a heart-rate value every 5 seconds), the user can plot the maximum, minimum, mean, etc. for every interval of 1 week, 1 day, 1 hour, etc. You can also choose different columns in the CSV file. This can be plotted in the form of a line plot, a bar plot, or a scattered plot, among others. The example in Figure 5 shows a box-plot of heart-rate every day.

Figure 5: In the master configuration dashboard, you can select different data and choose various graphs and different plot options to plot.


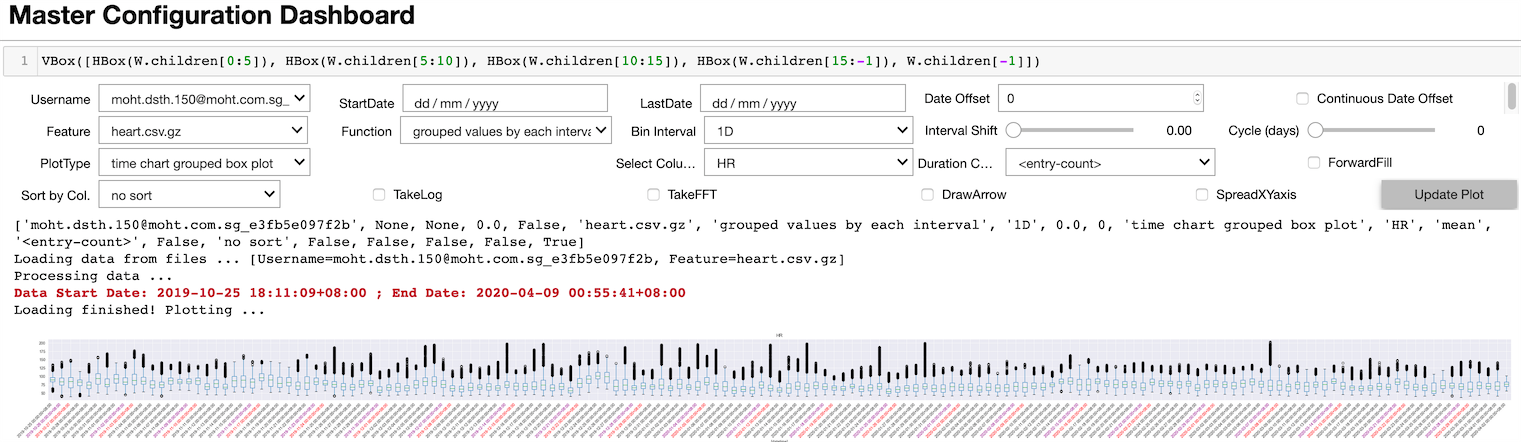


The main advantage of this toolkit is that it is highly customizable. In practice, users often want to display a fixed set of specific graphs on some specific data. Instead of clicking through various control items in the *master configuration dashboard* every time, they only need to do that once, copy and paste the configuration parameters (in Figure 5 immediately below the *Update Plot* button) into their custom scripts when calling the *draw* function. An example is shown in the overview dashboard Figure 6. This toolkit is designed to be general-purpose. The user can also manually load individual CSV files, not necessarily from this study, and visualize them. The only requirement on the CSV files is that it must contain a column called *timestamp* or *datetime*.

Figure 6: The overview dashboard, showing a particular patient’s data in many feature aspects over a period of time.


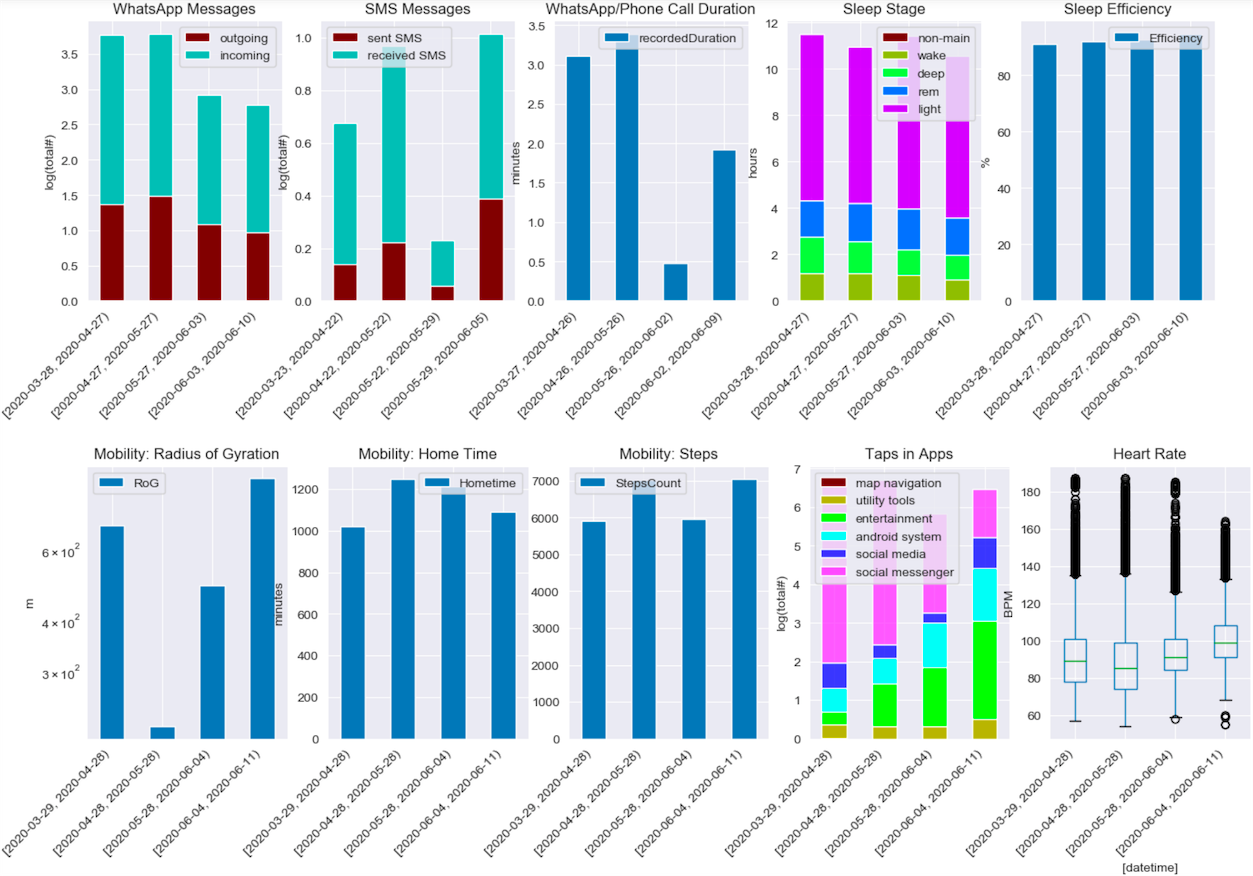


1. **Miscellaneous Platform Improvement**

We have also modified the system so that a clinician’s number can be set in the study settings. If it is not set in the study settings, then a ‘*PhoneNumberEntryActivity’* page will pop-up during the phone’s registration. Since some studies may have participants that have already consented, they should not need to consent again in the app. In the study settings, therefore, one may leave the ‘*text that will appear in the app’s consent form’* field empty, in which case the app will skip the consent page during the phone’s registration. In the prototype backend, the administrator/researcher can also add a remark for every participant. The color of the ‘*Last Upload Time’* column can be customized in the study settings via a small Python script. The same applies to the ‘*Data Completion Status’* column. Moreover, clicking on a particular participant’s ‘*Data Completion Status’* entry will produce an in-page pop-up window showing a table of the number of data records for that participant, for every feature, on every day. An example is shown in Figure 7.

Figure 7: Prototype back-end console showing the data completion details of the selected participant for every feature on every day


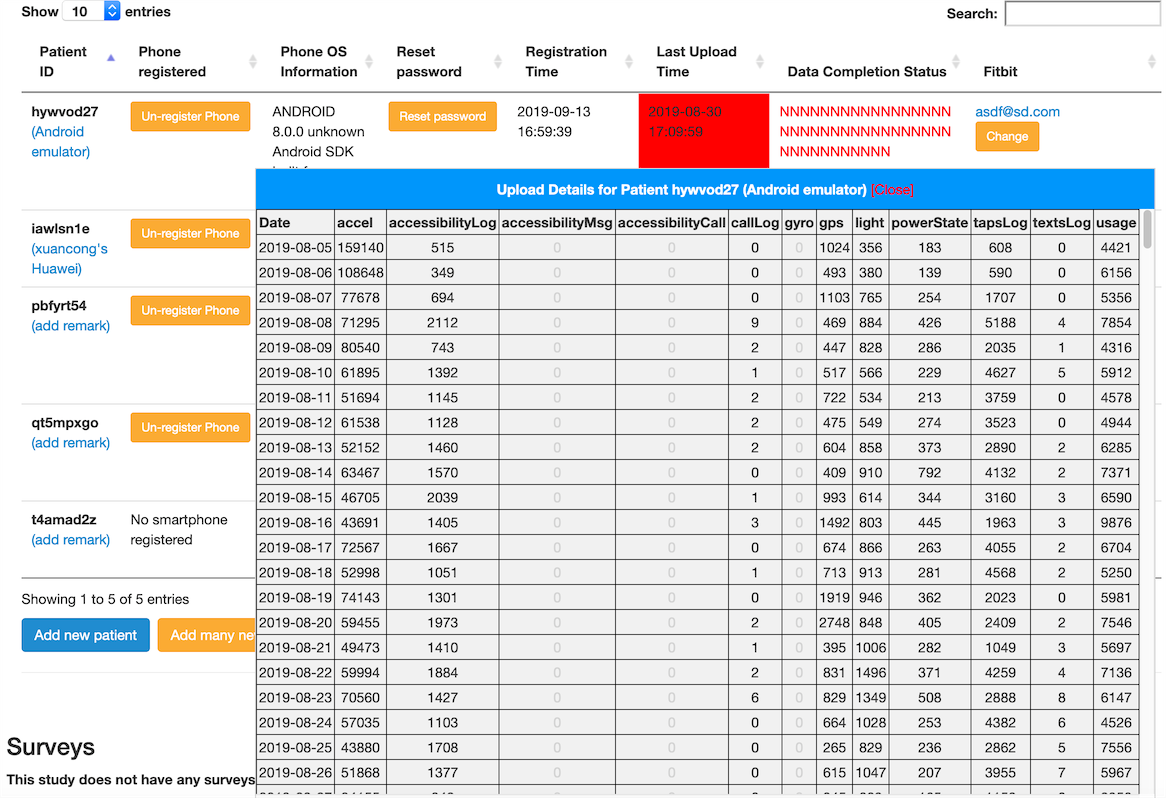


1. **Dashboards**

**8.1 Data Collection Dashboard**

This section gives a detailed explanation on what are the fields shown on the data collection dashboard.

From the phone:

1. *Location (last recorded hours ago)*: Time elapsed since the last location recorded until now
2. *Sociability (last recorded hours ago)*: Time elapsed since the latest WhatsApp message/WhatsApp call/SMS or a call
3. *Taps in Apps (last recorded hours ago)*: Time elapsed since the last touch on any application of the phone
4. *Last HOPES Uploaded*: Last time the system received data from the phone

From the Fitbit:

1. *Last Fitbit Uploaded*: Last time the system received data from the Fitbit
2. *Sleep (last uploaded hours ago)*: Time elapsed since the latest awake time after the last sleep

Other fields used in the dashboard:

1. *Participant*: Anonymous identifier given to the participant
2. *Last Clinic Visit (visit #)*: Last clinic visit day with the visit number
3. *Avg Fitbit Wearing Per Day (since last visit)*: Average Fitbit wearing time since the last visit, by using the heart rate which is captured in every 5 seconds. Time window for the calculation is from midnight of the clinic visit day to midnight of the day before the current day.
4. *Payment Progress (since last visit)*: Calculated based on the total Fitbit wearing time from the midnight of the last clinic visit day to the dashboard generation time. A day is considered as 22 hours long based on the requirement that a person should wear the Fitbit for at least 22 hours (i.e., this is considered 100% wearing-time). Payment progress refers to the participant’s progress towards an inconvenience payment that may be provided to some participants in a clinical trial.
5. *Phone Model*: Model of the phone
6. *Enrollment*: Onboarding date of the participant

Color codes:

To highlight the *issues* in *data-health*, a color code is used to make it easy to spot.

- *Green*: Normal state. Latest data received within the last 24 hours
- *Orange*: Standby state. Latest data received within the last 96 hours
- *Red*: Alert state. No data received within the last 96 hours

Issue summary:

Data-health issues that are red and orange are shown based on the issue type which are *Phone Sync Issues, Fitbit Sync Issues, Sleep Data Issues*

Other fields:

- *Last Fitbit Data Downloaded*: The last time the system downloaded Fitbit data from the Fitbit cloud
- *Dashboard Generated*: Time of the current view of the dashboard generated

**8.2 Clinician Dashboard**

This section describes the features used in the clinician dashboard in detail

***Sleep****:* The sleep graph is drawn based on the sleep duration (total time in bed) and sleep efficiency (ratio between sleep time and time in bed) directly retrieved from Fitbit. The vertical axis shows the sleep duration in hours while the horizontal axis indicates the duration for which the graph is drawn (see description of CW, PW, and PM below). The color of the graph depicts the sleep efficiency.

***Sociability****:* The total number of SMS/WhatsApp message exchanges and GSM/WhatsApp calls which are longer than 1 minute. The number of messages exchanged is the number of “different” contacts with which a person had exchanged messages during a day (midnight is the cut-off). The number of calls > 1 min means the number of times a person had calls longer than 1 minute during a day (midnight is the cut-off). The vertical axis shows the total number of messages exchanged and the number of calls > 1 min, and the horizontal axis shows the duration for which the graph is drawn (see description of CW, PW, and PM below).

***Mobility****:* Calculated based on the obfuscated GPS data, to describe the “movement” behavior of the user, considering home as the origin. Home is defined as the GPS location where the person has spent most of the time during the night (between 9 pm and 6 am). The mobility graph is drawn based on the time away from home and the radius of gyration, which are both calculated based on the GPS data. The vertical axis shows the time away from home in hours while the color of the graph indicates the average radius of gyration in kilometers. As shown in Figure 8, these features are calculated by taking the average in following time frames:

- CW – Current Week (7 days before 0000 hours today)
- PW – Past Week (7 days before the current week)
- PM – Past Month (30 days before the past week)

Figure 8: Illustration of the calculation of time periods

30 days

7 days

7 days

12 midnight

Past Month

Past Week

Current Week

Today

**8.3 Example Scenarios Presented in the Clinician Dashboard**

*Scenario 1: as shown in Figure 9a, an exemplary working adult of age 35, goes to the office, actively associates with friends, has kids and is married, generally has 6 hours of good sleep, often takes calls, sends messages to communicate with friends and colleagues, commutes to the office during the weekdays and goes out during the weekend.*

*
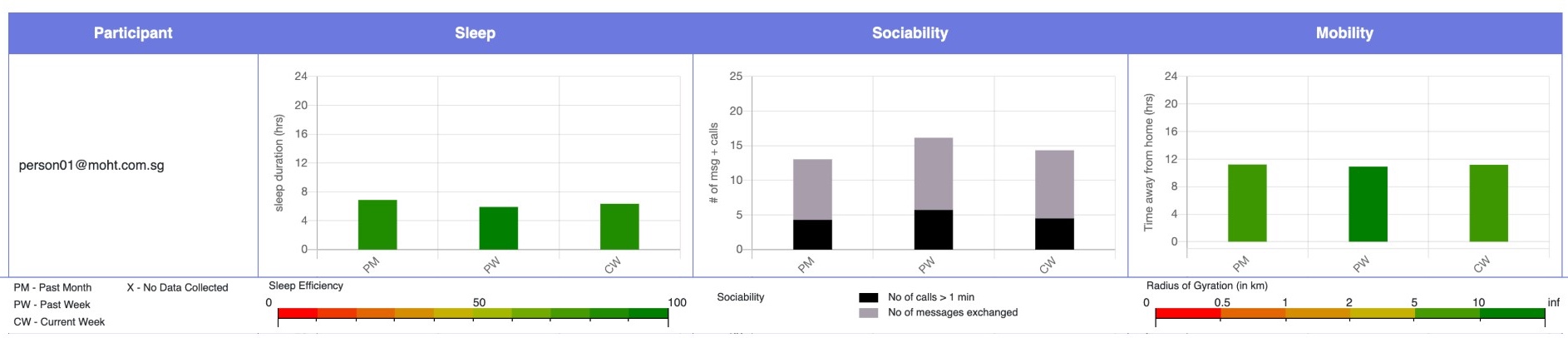
*

Figure 9a: example scenario 1 for the clinician dashboard

*Observations*: Consistent sleep durations and efficiency. No significant variations in number of calls and messages. Due to the regular behavior, time away from home is consistent and small variations in the radius of gyration occurs due to the places visited during the weekends

*Scenario 2: An exemplary participant with schizophrenia is living with parents and aged in the mid-20s, not going out often and plays video games at home. The participant doesn’t have many friends and has few social interactions, sleeps a lot. This participant may be suffering from a relapse.*

*
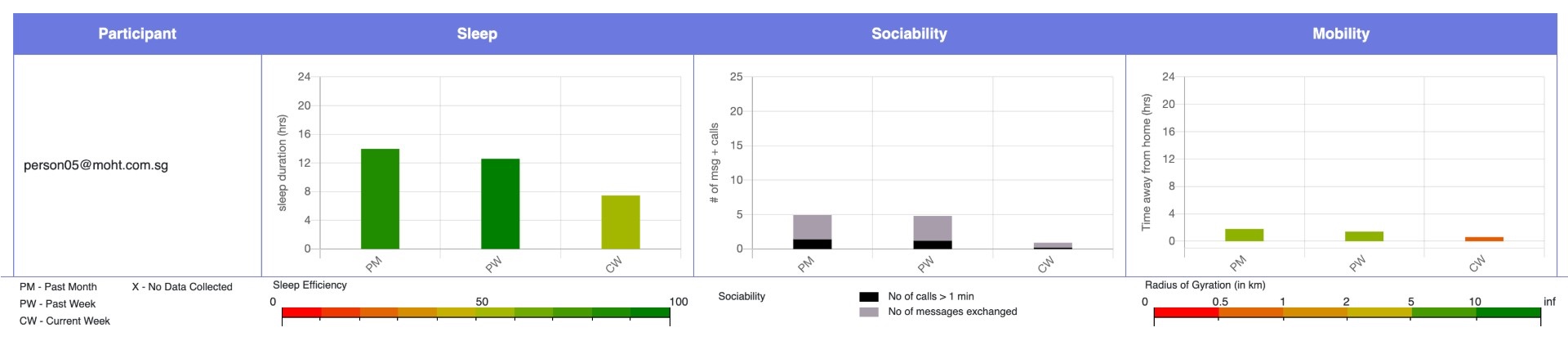
*

Figure 9b: example scenario 2 for the clinician dashboard

## *Observations:* Sleep duration is high compared to a working adult. Similarly, sociability is low compared to a working adult as he doesn’t have many friends. Since he preferers to stay at home and play video games, mobility is low compared to a working adult. But because of the possible emerging relapse during the last week, total sleeping hours and sleep efficiency has dropped. Because of pandemic restrictions, this participant may be isolating himself, and sociability and mobility have have dropped significantly.

**8.4 Data Completion Dashboard**

The *data completion dashboard* (see Figure 10) shows the historical completion status of the actual decrypted data from every participant over a customizable period (default is the last 90 days). It is implemented as a component module in the data processing pipeline and thus is updated every time when the data processing pipeline updates.

Figure 10: Data completion dashboard showing the completeness of every participant’s daily data.


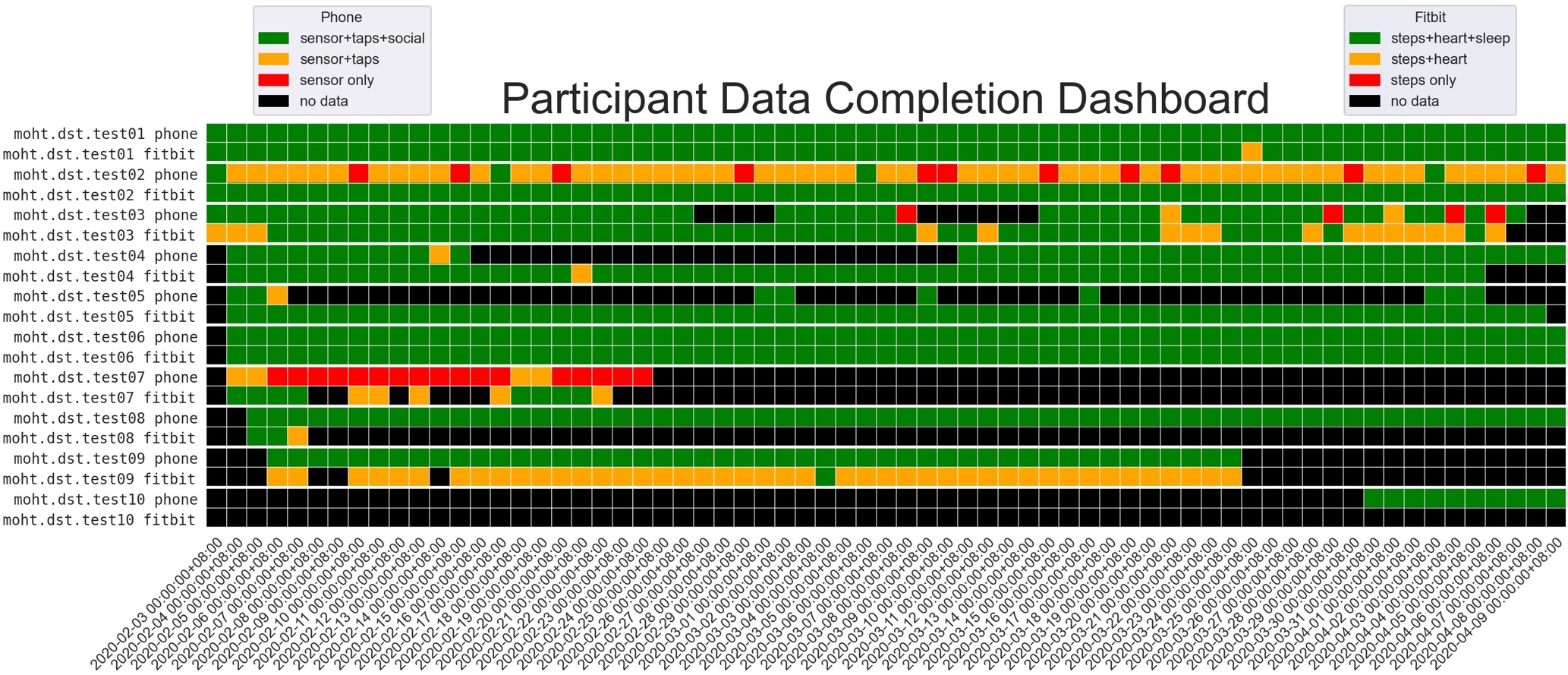


**8.5 Features Selected in Anomaly Detection Dashboard**

We have fitted several univariate time-series models to each of the following 12 *daily* digital phenotyping features; these were motivated by our HOPE-S study:

- sleep mean efficiency - the mean of the sleep efficiency scores during all periods of sleep;
- sleep tot hrs - the total amount of time spent (in hours) asleep;
- # steps - the total number of steps taken throughout the day;
- # walks - the total number of consecutive periods of steps (sampling interval is one minute), which we define as a *walk*;
- steps / min walk - the rate (in steps per minute) during all periods of a walk;
- social # sent - the number of messages and images sent on WhatsApp;
- social # recv - the number of messages and images received on WhatsApp;
- social # contact exch - the number of unique contacts that the participant both sent and received at least one message in WhatsApp;
- # taps - the number of taps in all apps;
- mean intap dur - the mean of the duration of intervals between all screen taps;
- RoG - the radius of gyration as measured by GPS and computed by the *Beiwe* backend;
- light mean lum - the mean recorded lumens by the ambient light sensor.
